# Steroids interfere with human carbonic anhydrase activity by using alternative binding mechanisms

**DOI:** 10.1080/14756366.2018.1512597

**Published:** 2018-09-17

**Authors:** Alessio Nocentini, Alessandro Bonardi, Paola Gratteri, Bruno Cerra, Antimo Gioiello, Claudiu T. Supuran

**Affiliations:** a Department NEUROFARBA – Pharmaceutical and nutraceutical section; Laboratory of Molecular Modeling Cheminformatics & QSAR, University of Firenze, Sesto Fiorentino, Italy;; b Department of NEUROFARBA, Pharmaceutical and Nutraceutical Section, University of Florence, Firenze, Italy;; c Department of Pharmaceutical Sciences, University of Perugia, Perugia, Italy

**Keywords:** Carbonic anhydrase, inhibitor, steroids, phenol, bile acid

## Abstract

Bile acids have been shown to inhibit human (h) carbonic anhydrases (CA, EC 4.2.1.1) along the gastrointestinal tract, including hCA II. The elucidation of the hormonal inhibition mechanism of the bile acid cholate to hCA II was provided in 2014 by X-ray crystallography. Herein, we extend the inhibition study to a wealth of steroids against four relevant hCA isoforms. Steroids displaying pendants and functional groups of the carboxylate, phenolic or sulfonate types appended at the tetracyclic ring were shown to inhibit the cytosolic CA II and the tumor-associated, transmembrane CA IX in a medium micromolar range (38.9–89.9 µM). Docking studies displayed the different chemotypes CA inhibition mechanisms. Molecular dynamics (MD) gave insights on the stability over time of hyocholic acid binding to CA II.

## Introduction

**Figure 1.**
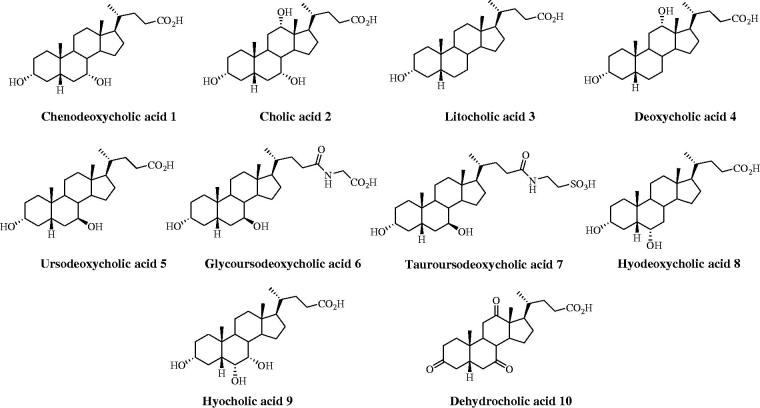
Structures of bile acids **1–10**.

**Figure 2.**
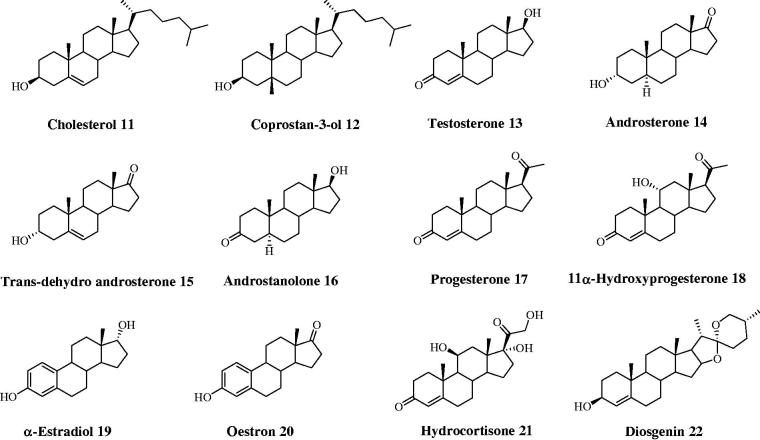
Structures of steroids **11–22**.

Steroids encompass a great variety of structurally related compounds that are widely distributed in the animal and plant kingdom^1^. The common chemical feature among the diverse classes is constituted by a perhydrocyclopentanophenanthrene nucleus. Steroids include crucial compounds for life as cholesterol, bile acids, and sex hormones that play several physiological responses mediated by both genomic and non-genomic actions^2^. A variety of natural and semi-synthetic steroids are used in therapy as anti-inflammatory, immunosuppressive, anabolic, and contraceptive agents, as well as for the prevention of coronary disease, and for the management of diabesity and declared AIDS. Remarkably, steroids are a wealthy source of therapeutic agents for specific forms of cancer^3^: indeed, they act as aromatase and sulfatase modulators against breast cancer, and as 5α-reductase and CYP17 inhibitors to treat benign prostatic hyperplasia and advanced prostate cancer, respectively. Additionally, semi-synthetic steroidal derivatives are very relevant for the discovery of chemical probes for exploring molecular mechanisms of action of understudied biological targets and pathways^4–6^. In this paper, we aim to determine the activity of a set of major naturally-occurring steroids (Figures 1 and 2) as carbonic anhydrase inhibitors (CA, EC 4.2.1.1).

Carbonic anhydrases consist in a superfamily of zinc enzymes which catalyze the reversible hydration of CO_2_ into HCO_3_ and protons by a metal hydroxide nucleophilic mechanism^7^^,^^8^. Seven genetically distinct CA families (α-, β-, γ-, δ-, ζ-. η- and θ-CAs.) are known to date^7–10^ .The 15 different α-CA isoforms isolated in humans (h) feature catalytic activity, sub-cellular localization and organ/tissue distribution. Among the catalytically active isoforms, some are cytosolic (CA I, CA II, CA III, CA VII and CA XIII), others are membrane bound (CA IV, CA IX, CA XII, CA XIV and CA XV), two of them are mitochondrial (CA VA and CA VB), and one isozyme is secreted in saliva (CA VI)^7^. A variety of human patho-physiological processes shows abnormal levels or activities of these enzymes, and this makes CA isozymes valuable targets for many pharmacological applications such as antiglaucoma drugs, diuretics, antiobesity, anticonvulsant and/or antitumor agents/diagnostic tools^7^. Several hCAs along the gastrointestinal tract, including hCA II, have been shown to be inhibited by the 5β-steroids bile acids^11^, the primary end-products of cholesterol catabolism, resulting in a damage of the gastric mucosa. The damage produced mainly by primary bile acids and their conjugates has been related with gastric mucosal CAs inhibition in rats and humans^12^. The structural evidence of the hormonal inhibition mechanism of the bile acid cholate to hCA II has been given in 2014^13^. The carboxylate was found to bind to the zinc ion in a bivalent manner displacing the zinc-bound solvent molecule.

Herein, we extend the inhibition study to a wealth of steroids against 4 relevant hCA isoforms. Beyond understanding the interference of the CA activity by steroids, the knowledge of the CA inhibition profiles of several such derivatives could be of interest and drive the design of new non-sulfonamide-like compounds, that can be easily transported across the cellular membrane.

## Experimental section

### Steroids

Chenodeoxycholic acid (**1**), ursodeoxycholic acid (**5**), hyodeoxycholic acid (**8**), hyocholic acid (**9**), coprostan-3-ol (**12**), *trans*-dehydroandrosterone (**15**), progesterone (**17**), 11α-hydroprogesterone (**18**), α-estradiol (**19**), and diosgenin (**22**) were purchased from Sigma-Aldrich. Cholic acid (**2**), lithocholic acid (**3**), deoxycholic acid (**4**), cholesterol (**11**), testosterone (**13**), and oestron (**20**) were purchased from Fluka. Dehydrocholic acid (**10**) and hydrocortisone (**21**) were purchased from Janssen Chimica and BDH Chemicals, respectively. Glyco (**6**) and tauroursodeoxycholic acid (**7**) were prepared as previously reported^14^^,^^15^. Purity of tested compounds **1**–**22** was >95%.

### Carbonic anhydrase inhibition

An Applied Photophysics stopped-flow instrument has been used for assaying the CA catalyzed CO_2_ hydration activity^16^. Phenol red (at a concentration of 0.2 mM) has been used as indicator, working at the absorbance maximum of 557 nm, with 20 mM Hepes (pH 7.4) as buffer, and 20 mM Na_2_SO_4_ (for maintaining constant the ionic strength), following the initial rates of the CA-catalyzed CO_2_ hydration reaction for a period of 10–100 s. The CO_2_ concentrations ranged from 1.7 to 17 mM for the determination of the kinetic parameters and inhibition constants. For each inhibitor at least six traces of the initial 5–10% of the reaction have been used for determining the initial velocity. The uncatalyzed rates were determined in the same manner and subtracted from the total observed rates. Stock solutions of inhibitor (0.1 mM) were prepared in distilled-deionized water and dilutions up to 0.01 nM were done thereafter with the assay buffer. Inhibitor and enzyme solutions were preincubated together for 15 min at room temperature prior to assay, in order to allow for the formation of the E-I complex. The inhibition constants were obtained by non-linear least-squares methods using PRISM 3 and the Cheng–Prusoff equation, as reported earlier^17–19^, and represent the mean from at least three different determinations. All CA isofoms were recombinant ones obtained in-house as reported earlier^20^^,^^21^.

### Computational studies

4E3H^26^ and 4PXX^13^ crystal structures was prepared according to the Protein Preparation module in Maestro – Schrödinger suite, assigning bond orders, adding hydrogens, deleting water molecules, and optimizing H-bonding networks^22^. Finally, energy minimization with a root mean square deviation (RMSD) value of 0.30 was applied using an Optimized Potentials for Liquid Simulation (OPLS_2005) force field. 3 D ligand structures were prepared by Maestro^22a^ and evaluated for their ionization states at pH 7.4 ± 0.5 with Epik^22b^ OPLS-2005 force field in Macromodel^22e^ was used for energy minimization for a maximum number of 2500 conjugate gradient iteration and setting a convergence criterion of 0.05 kcal mol^−1 ^Å^−1^. The docking grid was generated using Glide^22f^ with default settings, with the center located on the center of mass of the cocrystallized ligand. Ligands were docked employing the standard precision mode (SP) retaining the best five poses of each molecule as output. The top ranked binding pose of each compound was then analyzed in terms of coordination, hydrogen bond interactions and hydrophobic contacts.

The best scored binding pose of **9** to the CA II active site was submitted to a MD simulation using Desmond^23^ and the OPLS2005 force field. Specifically, the system was solvated in an orthorhombic box using TIP4PEW water molecules, extended 15 Å away from any protein atom. Then, it was neutralized adding a concentration of 0.15 M chlorine and sodium ions. The simulation protocol included a starting relaxation step and a final production phase of 10 ns. In particular, the relaxation step comprised the following: (a) a stage of 100 ps at 10 K retaining the harmonic restraints on the solute heavy atoms (force constant of 50.0 kcal mol^−1 ^Å^−2^) using the NPT ensemble with Brownian dynamics; (b) a stage of 12 ps at 10 K with harmonic restraints on the solute heavy atoms (force constant of 50.0 kcal mol^−1 ^Å^−2^), using the NVT ensemble and Berendsen thermostat; (c) a stage of 12 ps at 10 K and 1 atm, retaining the harmonic restraints and using the NPT ensemble and Berendsen thermostat and barostat; (f) a stage of 12 ps at 300 K and 1 atm, retaining the harmonic restraints and using the NPT ensemble and Berendsen thermostat and barostat; (g) a final 24 ps stage at 300 K and 1 atm without harmonic restraints, using the NPT Berendsen thermostat and barostat. The final production phase of MD was run using a canonical the NPT Berendsen ensemble at temperature 300 K. During the MD simulation, a time step of 2 fs was used while constraining the bond lengths of hydrogen atoms with the M-SHAKE algorithm. The atomic coordinates of the system were saved every 50 ps along the MD trajectory. The occupancy of intermolecular hydrogen bonds and hydrophobic contacts was calculated along the production phase of the MD simulation with the Simulation Interaction Diagram tools implemented in Maestro. MD snapshots were clustered with the script Cheminformatics – Clustering of Conformers from Schrodinger, using the average linkage clustering method based on the RMSD matrix between the conformers Cartesian coordinates – non hydrogen atoms only.

## Results and discussion

### Biological activity

Among the twelve catalytically active hCAs, the isoforms chosen for our studies involved the cytosolic hCA I and II (involved in a host of physiologic processes)^7^, the membrane-bound hCA IV (involved in glaucoma, retinitis pigmentosa, stroke and rheumatoid arthritis)^24^^,^^25^ and the tumor-associated hCA IX (abundant in hypoxic tumors and recently validated as antitumor target)^26^^,^^27^.

Inhibition data of steroids **1–22** against hCA I, II, IV and IX were measured by a stopped flow CO_2_ hydrase assay and are shown in Table 1^16^. Acetazolamide, a clinically used sulfonamide inhibitor, was used as standard.

**Table 1.** Inhibition data of human CA isoforms hCA I, II, IV and IX with compounds reported here and the standard sulfonamide inhibitor acetazolamide (**AAZ**) by a stopped flow CO_2_ hydrase assay.

	K_I_ (µM)*			
Compound	hCAI	hCA II	hCA IV	hCA IX
Chenodeoxycholic acid (**1**)	>100	53.9	>100	63.0
Cholic acid (**2**)	>100	48.9	>100	47.1
Lithocholic acid (**3**)	>100	64.5	>100	70.2
Deoxycholic acid (**4**)	>100	51.0	>100	55.0
Ursodeoxycholic acid (**5**)	95.9	71.4	>100	73.1
Glycoursodeoxycholic acid (**6**)	>100	78.4	>100	89.9
Tauroursodeoxycholic acid (**7**)	>100	82.9	>100	42.9
Hyodeoxycholic acid (**8**)	>100	58.4	>100	67.7
Hyocholic acid (**9**)	83.3	38.9	>100	71.9
Dehydrocholic acid (**10**)	>100	57.8	>100	53.9
Cholesterol (**11**)	>100	>100	>100	>100
Coprostan-3-ol (**12**)	>100	>100	>100	>100
Testosterone (**13**)	>100	>100	>100	>100
Androsterone (**14**)	>100	>100	>100	>100
*Trans*-dehydroandrosterone (**15**)	>100	>100	>100	>100
Androstanolone (**16**)	>100	>100	>100	>100
Progesterone (**17**)	>100	>100	>100	>100
11α-Hydroxyprogesterone (**18**)	>100	>100	>100	>100
α-Estradiol (**19**)	87.8	40.4	>100	49.6
Estron (**20**)	>100	50.8	>100	71.4
Hydrocortisone (**21**)	>100	>100	>100	>100
Diosgenin (**22**)	>100	>100	>100	>100
AAZ	0.25	0.012	0.074	0.025

* Mean from 3 different assays, by a stopped flow technique (errors were in the range of ±5–10% of the reported values).

The data of Table 1 show that a basic requirement to address a though weak inhibitory efficacy to steroidal derivatives is the presence of a functional moiety that plays the role of zinc-binding or anchoring group to the metal-coordination center, i.e. carboxylates, sulfonates and phenols. Unlike this latter, it should be stressed that analog derivatives bearing OH moieties of the aliphatic type do not exhibit any inhibitory efficacy.

Nevertheless, the cytosolic hCA I and the membrane-bound hCA IV are inhibited by none of the assayed derivatives below 100 µM, except steroids **5**, **9** and **19** which feebly affect hCA I activity with inhibition constants (K_I_s) of 95.9, 83.3, 87.8 µM, respectively.

The ubiquitous hCA II and tumor-associated hCA IX were comparably inhibited by carboxylic acids **1–6**, **8–10**, sulfonic acid **7** and phenols **19**, **20** in the micromolar range spanning between 38.9 and 89.9 µM. Tauroursodeoxycholic acid (**7**) stands out as the most efficient hCA IX inhibitor, being instead its action the least efficient against hCA II. Repositioning of the alcoholic moieties mainly located at the outer edge of the molecular structures has been shown to slightly alter the weak inhibition profiles. The lengthening of the carboxyalkyl chain of ursodeoxycholic acid (**5**) by a glycine unit as in **6** does not affect the derivatives efficacy against both considered isoforms. Reduction of the enone system at ring A of testosterone (**13**) to 5α-steroids androstanolone (**16**) and androsterone (**14**) does not interfere with the hCA II and IX inhibitory efficacy.

### Computational studies

**Figure 3.**
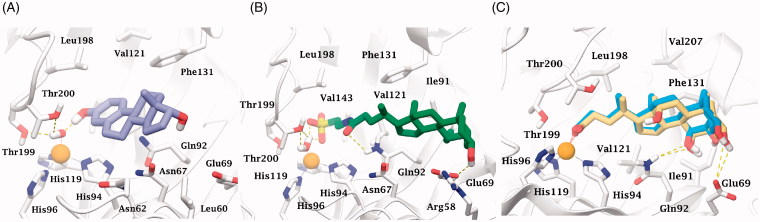
Dockings of (A) α-estradiol (**19**) and (B) tauroursodeoxycholic acid (**7**) within hCA II. (C) Superposed docked hyocholic acid (**9**) (blue) and cholic acid (**2**) (yellow) X-ray solved orientation within hCA II.

According to the binding mechanism of the different class of CAI (phenols, sulfonates, and carboxylates) herein studied^28–35^, docking simulations were carried out on representative steroids with hCA II, namely **7**, **9**, **19**. Sulfonate (**7**) and compounds bearing a phenol group (**19**, **20**) act as zinc-bound nucleophile anchoring group, whereas carboxylates (**1–6**, **8–10**) bind directly to the catalytic Zn ion (zinc binders). It was found that the phenolic OH of α-estradiol (**19**) is H-bonded to the zinc-bound hydroxide ion, that is in turn stabilized by three other H-bonds, acting as donor to Thr199 and Thr200 side chain OH and as acceptor with the Thr199 backbone NH. Noteworthy, the phenolic portion of α-estradiol anchors to the nucleophile locating more externally than cocrystalized simple ligands such as hydroquinone (PDB code 4E3H), owing to the steric hindrance lined by the tetrahydrophenantrenic core. This latter is involved in π-π and π-alkyl interactions with Leu198, Thr200, Phe131, Val121, His94, Gln92, Asn67, Asn62, and Leu60 side chain. Furthermore, the docked pose features an H-bond between the alcoholic moiety of **19** and the Asn67 side chain carbonyl group. The terminal sulfonate group of tauroursodeoxycholic acid (**7**) anchors to the pseudo-tetracoordinated zinc-bound water molecule by a five H-bonds network involving the ligand, the nucleophile and the enzyme (Thr199 and Thr200). Further H-bonds stabilize the docked pose. The carboxy amidic moiety acts as acceptor by the amidic NH_2_ of Gln92, the alcoholic function in C_7_ position acts as donor to Ile91 and Phe70 backbone carboxy and NH group respectively and the C_3_-OH donates a H-bonds to Glu69 side chain. Like **19**, the tetrahydrophenantrenic ring of **7** was involved in Van der Waals interactions with Val143, Val207, Trp209, Leu198, Thr199, Thr200, His94, His119, Gln92, Phe131, Val121, Ile91, Glu69, and Arg58 residues (Figure 3).

According to the X-ray solved structure (PDB code 4PXX) of cholic acid (**2**) in complex with hCA II^13^, the carboxylic group of the docked hyocholic acid (**9**) directly coordinates the zinc ion in a bidentate manner, and showing a very good agreement with the positioning of the crystallographic complex ligand. Thorough analogies between cholic acid (**2**) crystallography and hyocholic acid (**9**) docking with hCA II were found. As in the X-ray solved structure, the coordination is further sustained by a H-bond occurring between the backbone NH of Thr199 and the carboxylate group. The alcoholic function in C_7_ accepts an H-bond from Gln92 side chain NH_2_, and further stabilization of the pose comes from hydrophobic interactions taking place between the tetrahydrophenantrenic core and His94, His96, His119, Thr199, Thr200, Leu198, Pro201, Val121, Gln92, Phe131 and Ile91 residues. Unlike cholic acid (**2**), the hydroxy group at C_3_ position of **9** is not in H-bond contact with Glu69.

**Figure 4.**
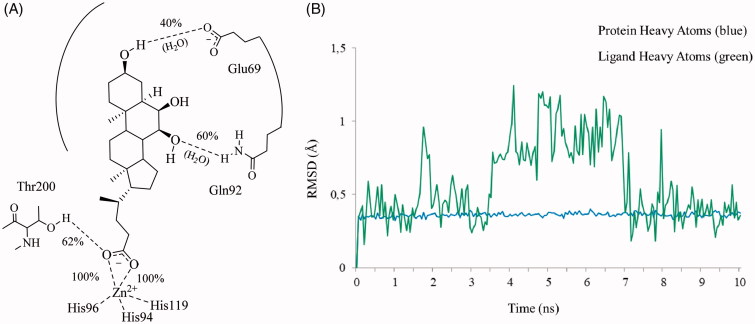
Analysis of the MD simulation of **9** docked to hCA II. (A) Coordination and H-bonds occupancies within 10 ns MD for **9 -** hCA II complex. (B) Rmsd representation of the heavy atoms of the receptor and the ligand from the starting model structure during the simulation.

**Figure 5.**
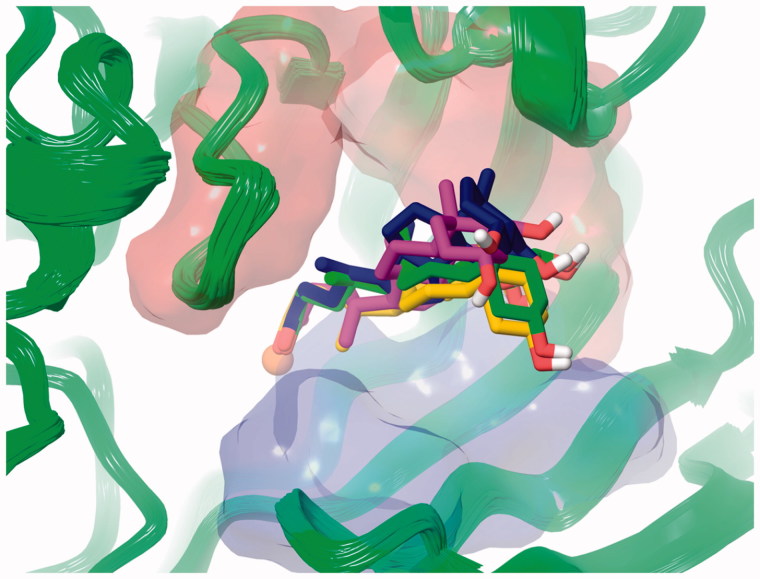
Superposed representative orientations of the four identified clusters within superposed protein backbones of 200 frames of MD.

**Figure 6.**
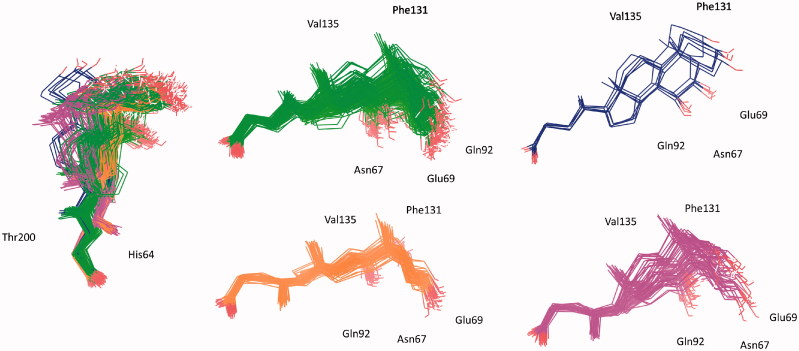
Conformer families of **9** identified over the 10 ns MD period.

The statistical stability of the docked pose of hyocholic acid (**9**) as well as the stability of the H-bonds network involving the hydroxy moieties has been studied by analyzing the ligand conformation and the positional changes upon 10 ns molecular dynamics (MD) simulation of **9** docked to hCA II, as starting point. The blue line in Figure 2(B) shows how the protein C-alphas evolve over the 10 ns MD period and it indicates that the system is equilibrated. The green line, representing the aligned-ligand RMSD (only heavy atoms), gives insights onto how compound **9** is stable with respect to the enzyme binding pocket. The bidentate coordination is stably maintained (Figure 4(A)). The H-bond that is established either directly or mediated by a water molecule between the C_7_-OH and the Gln92 side chain NH_2_ displayed a 60% stability over 10 ns, whereas analog interaction taking place between the C_3_-OH and Glu69 side chain is maintained for 4 ns (40% of the overall dynamics simulation time) (Figure 4(B)). The inspection of the MD trajectory points out interesting conformational transitions taking place in between 3 and 7 ns of the overall 10 ns time scale. Cluster analysis may help in identifying the relevant internal fluctuations which occur within the ligand structure during the simulation (Figures 5 and 6). Four main conformer families were found which substantially shares the zinc binding mode and differ for the orientation of the methyl group on C21 (orientation A and B according to the side - His64 or Thr200 - towards which the methyl group points to) and the hydrophobic or hydrophilic portion of hCA II occupied by the polycyclic scaffold (Figures 5 and 6). The percentage (about 60%) of time spent by **9** in the docking found conformation testifies the stability of the pose.

## Conclusions

Bile acids are known to inhibit hCAs along the gastrointestinal tract, including hCA II^11^. The structural elucidation of the hormonal inhibition mechanism of the bile acid cholate to hCA II was furnished in 2014. The present study reports inhibition studies of physio-pathologically relevant CAs with a set of selected steroids. Steroids displaying pendants and functional groups of the carboxylate, phenolic or sulfonate types appended at the tetracyclic ring were shown to inhibit the cytosolic CA II and the tumor-associated, transmembrane CA IX in a medium micromolar range. Since the aforementioned functional groups are known to exert a CA inhibitory action by different binding mechanism, computational studies on representative derivatives were undertaken. A 10 ns MD gave insights on the stability of hyocholic acid (**9**) binding to CA II. Beyond understanding the interference of the CA activity by steroids, the knowledge of the CA inhibition profiles of such derivatives could be of interest and drive the design of novel steroidal non-sulfonamide-like CA inhibitors.
